# Analysis of patients’ privacy and associated factors in the perioperative period

**DOI:** 10.3389/fmed.2023.1242149

**Published:** 2023-10-11

**Authors:** Mingyang Tan, Hongyu Li, Xiaofei Wang

**Affiliations:** ^1^Department of Nursing, Jinzhou Medical University, Jinzhou, China; ^2^Institute of Medical Education, Jinzhou Medical University, Jinzhou, China

**Keywords:** perioperative, privacy, influence factors, surgery, cross-sectional survey

## Abstract

**Background:**

Healthcare professionals recognize how to protect patient privacy in order to effectively reduce the occurrence of conflict between the two parties. Therefore, understanding the protection of patient privacy during the perioperative period and the relevant factors affecting privacy is essential to improving healthcare delivery.

**Methods:**

This was a cross-sectional survey of a convenience sample of 400 perioperative patients. General demographic information, the perioperative privacy scale (PPS), and the Amsterdam preoperative anxiety and information scale (APAIS) were used for the survey. And factors affecting patient privacy were investigated by ANOVA or t-test analysis, Pearson correlation analysis, and linear regression models.

**Results:**

This study found that perioperative patient privacy satisfaction scores were (53.51 ± 12.54). The results of the univariate analysis showed that factors affecting privacy satisfaction included gender, age, and the number of surgeries (*p* < 0.05). Preoperative anxiety and Information Needs Scale was negatively associated with the perioperative patient privacy satisfaction (*r* = −0.807, *p* < 0.01). Further analysis was performed using linear regression models to finally obtain five factors affecting perioperative patient privacy: gender, age, anesthesia modality, the number of surgeries, and the Amsterdam preoperative anxiety and information.

**Conclusion:**

Healthcare professionals working in healthcare facilities need to be aware of the sensitivity of different populations to privacy when protecting patient privacy. Patients’ preoperative anxiety and information need status affect privacy satisfaction. This will mean that healthcare professionals will be able to identify key privacy concerns early and take appropriate action.

## Introduction

The patient’s right to privacy refers to the patient’s secrets that were legally obtained by medical personnel and institutions due to consultation and treatment needs but could not be disclosed through illegal means, in the process of receiving medical services at the time of medical treatment (1). Protecting patient privacy was an ethical obligation of medical personnel, not only protected by law but also a part of the medical process that cannot be ignored (2). With the continuous improvement of laws and regulations in various countries and the increasing awareness of individual rights, the status of privacy had gradually increased in the minds of citizens. In order to be able to optimize the medical treatment environment, it was necessary to ensure the privacy of the patient during the procedure. Therefore, it was of utmost importance to investigate the importance of patient privacy so that the current public health environment can be improved.

Privacy protection was a central tenet of the doctor-patient safety relationship (3). Research showed that protecting patients’ privacy is high on the list of medical professional values (4). Public health care workers should respect patients’ privacy and continuously realize professional values. However, the insufficient protection of patient privacy or even invasion thus leads to the gradual prominence of the doctor-patient conflict (5, 6), and surgery was the most direct way to expose patient privacy (7). Ozturk et al. (8) found that 43.9% of nurses believed that patients’ privacy was violated during medical visits. Excessive exposure to patient privacy during surgery could lead to negative emotions such as mistrust (9), which reduces the effectiveness of treatment and increases the incidence of doctor-patient disputes (10). Therefore, understanding the extent of patient privacy protection in China is essential to improve satisfaction with healthcare services (11, 12).

As healthcare professionals, increasing awareness of safety and risk in the perioperative environment was critical and can significantly reduce the incidence of adverse events, which in turn can impact the desired goals of patients throughout their treatment (13). In order to ensure an intimate relationship between patients and medical staff, their right to privacy must be respected (12, 14, 15). Dialog with patients in the perioperative period could build trust between the two sides (16, 17) and make patients gain a complete sense of security (18), thus alleviating patient tension and anxiety to form an excellent doctor-patient atmosphere.

One study (19) showed that to some extent patient preoperative anxiety is the most common negative emotion in the perioperative period. Stress and anxiety could stay with the patient from the confirmation of surgery until the end of the procedure. Excessive negative emotions of anxiety could lead to a poor surgical experience for the patient and increase the occurrence of doctor-patient conflicts. Strengthening trust between doctor and patient is a protective factor against preoperative anxiety when the patient’s information needs are met (20).Preoperative anxiety is common in perioperative patients (21), and patient privacy satisfaction can be improved by enhancing perioperative education and interventions.

Negligence in the protection of patient privacy seriously affects the patient’s experience during the visit and the quality of services. Focusing on and improving perioperative patients’ privacy satisfaction thereby improving the quality of health care services. Some research has been conducted on the prevalence and characteristics of patient electronic information privacy. However, the factors associated with perioperative patients have not been adequately investigated. Therefore, further studies are needed to comprehensively analyze perioperative patient privacy and its possible correlates. The purpose of this study was to assess the privacy satisfaction of Chinese patients and the factors influencing it using a general demographic questionnaire, a perioperative patient privacy questionnaire, and a preoperative anxiety and information needs questionnaire. The following research questions were posed: sociodemographic information, preoperative anxiety and information needs are influential factors affecting perioperative patients’ privacy satisfaction.

## Methods

### Study design and sample size

This was a cross-sectional study, in which patients in the perioperative period of 2 hospitals in Zibo City, Shandong Province, were selected by convenience sampling method from February to March 2023 as study subjects. Inclusion criteria: (1) age ≥ 18 years; (2) in patients in the perioperative period of surgical procedures; (3) informed consent and voluntary participation in the study; (4) cognitive ability to correctly understand the questionnaire content. Exclusion criteria: (1) suffering from psychiatric disorders; (2) surgery time < 1 h; (3) those who withdrew in the middle of the procedure, (4) patients with language and communication disabilities. G-Power 3.1.9.2 was used to calculate the sample size in the study, based on an α level of 0.05, a power of 0.80, and a medium effect size of 0.25, was adequate to identify significant effects. In this study, a total of 407 questionnaires were distributed, of which 400 were valid, with an effective recovery rate of 98.3%.

## Instruments

### The general information questionnaire

Patient’s gender, age, education, marital status, anesthesia modality, clinic, type of room, and the number of surgeries(times) were collected.

### The perioperative privacy scale

The Perioperative Privacy Scale was developed by Yayla et al. (22) in Turkey to assess patients’ privacy during the perioperative period. In 2023 Tan et al. (23). translated this scale into a Chinese version of the PPS scale after a scientific translation process, which had good reliability and validity. The Chinese version of the Perioperative Patient Privacy Scale was used to assess patients’ privacy satisfaction during the perioperative period. The scale includes three dimensions of preoperative privacy (7 items), intraoperative privacy (3 items), and postoperative privacy (6 items), with a total of 16 items. Items were rated on a 5- level Likert scale from 1 to 5, corresponding to a scale from completely disagree to agree completely. In this study, Cronbach’s alpha coefficient of the scale was 0.939.

### The Amsterdam preoperative anxiety and information scale

This scale was developed by Moerman et al. (24) in 1995 to assess the psychological anxiety and information needs of patients in the preoperative phase. In 2017, Le (25) established a Chinese version of the scale, which has good reliability with a Cronbach’s alpha coefficient of 0.815. The scale consists of 6 items divided into 3 dimensions (anesthesia-related factors, surgery-related factors, and information need factors). The scale consists of 6 items, each of which is rated on a scale of 1 to 5, i.e., “1” is “not at all” to “5” is “very.” “The higher the score of an entry, the more it corresponds to the state of the surgical patient. In this study, Cronbach’s alpha coefficient of the scale was 0.929.

### Data collection

The researcher contacted the hospital nursing department and conducted the study in each department with consent. The significance of the purpose of the study and precautions were explained to the subjects through a face-to-face survey, and their informed consent was obtained in writing.

### Statistical analyses

Data analysis was performed using the IBM SPSS26.0 and GraphPad Prism 9 statistical software. All data were collected and checked by two investigators independently. All data conform to a normal distribution. A descriptive study was conducted for demographic characteristics, and the scores of each scale were described using means and SD. To analyze the differences, the t-test and the ANOVA test were performed. The results of the correlation analysis were completed and displayed by R (V4.0.2). Pearson correlation analysis was performed for APAIS and PPS metrics using the R (V4.0.2) corrplot package, and these metrics were significantly correlated at *p* < 0.05 when *R* > 0.5 or *R* < −0.5. Linear regression analysis was conducted to find independent factors associated with patient privacy. The VIF for each covariate was below 5, a result considered acceptable. The results were considered statistically significant at *p* < 0.05.

### Ethical considerations

This study was approved by the Ethics Committee of Jinzhou Medical University(no.JZMULL2022008). As a prerequisite for participation, written informed consent was obtained from the participants or their legal representatives. The study was conducted in accordance with accepted ethical standards.

## Results

### Participant’s characteristics and their univariate analysis

The 400 perioperative participants included 195 male participants (48.8%) and 205 female participants (51.3%). 47% were between the ages of ≥60 years; the vast majority of participants were married (74.3%). The results of the ANOVA test showed significant differences in the privacy status of the perioperative patients in terms of gender, age, anesthesia modality, department, and number of surgeries. The overall characteristics can be seen in Table 1.

**Table 1:** The distribution of the patients by their socio-demographic and Clinical characteristics (*N* = 400).

Characteristics	*n* (%)	PPS	*t*/*F* value	*p* value
*Gender*				
Male	195 (48.8)	55.48 ± 12.05	3.089	0.002
Female	205 (51.3)	51.64 ± 12.74		
*Age (years)*				
18–44	59 (14.8)	47.56 ± 14.62	8.088	<0.001
45–59	153 (38.3)	54.63 ± 11.00		
≥60	188 (47.0)	54.47 ± 12.56		
*Education*				
Junior	131 (32.8)	53.05 ± 13.60	0.648	0.584
Middle	108 (27.0)	52.57 ± 11.83		
High school	110 (27.5)	54.75 ± 12.40		
College and above	51 (12.8)	54.02 ± 11.51		
*Marital status*				
Married	297 (74.3)	53.02 ± 12.75	0.928	0.396
Single	56 (14.0)	55.25 ± 13.06		
Others	47 (11.8)	54.55 ± 10.32		
*Anesthesia modality*				
General	107 (26.8)	51.53 ± 13.19	4.149	0.016
Local	110 (27.5)	56.25 ± 11.66		
Others	183 (45.8)	53.03 ± 12.44		
*Clinic*				
General surgery	73 (18.3)	57.34 ± 11.54	3.658	0.006
Cardiothoracic surgery	69 (17.3)	54.99 ± 11.41		
Orthopedic	72 (18.0)	54.15 ± 12.52		
Urology	46 (11.5)	50.85 ± 11.19		
Others	140 (35.0)	51.34 ± 13.48		
*Type of room*				
Single	69 (17.3)	54.00 ± 11.89	0.970	0.380
Double	145 (36.3)	54.46 ± 12.69		
Triple	186 (46.5)	52.59 ± 12.65		
*Number of surgeries(times)*				
0	234 (58.5)	52.23 ± 13.03	3.869	0.022
1	135 (33.8)	55.93 ± 11.71		
≥2	31 (7.8)	52.65 ± 10.97		

### PPS scores for perioperative patients

**Figure 1:**
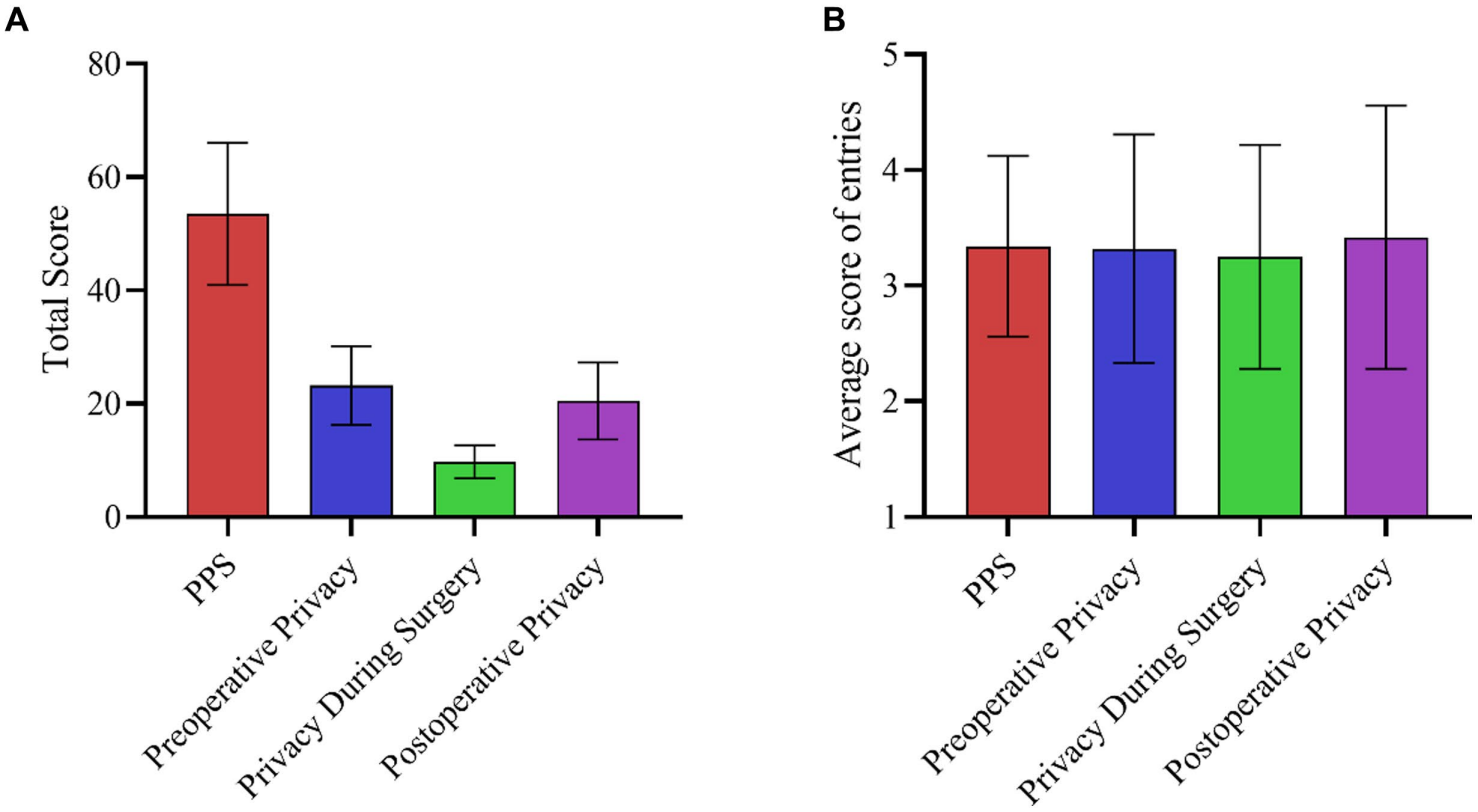
PPS scores for perioperative patients. **(A)** PPS scale and dimensional total scores; **(B)** PPS scale and mean scores for each dimensional entry.

The mean (SD) score for PPS was (53.51 ± 12.54), the mean (SD) preoperative privacy score was (23.26 ± 6.93), the mean (SD) privacy during surgery score was (9.75 ± 2.90), and the mean (SD) postoperative privacy score was (20.50 ± 6.83). Perioperative patients and their privacy scores for each dimension are shown in Figure 1.

### APAIS scores for perioperative patients

The mean (SD) score for APAIS was (16.66 ± 6.15), for anesthesia-related factors (2.79 ± 1.20), for surgery-related factors (2.87 ± 1.20), and for information needs factors (2.68 ± 1.09).

### Correlation analysis of privacy and the Amsterdam preoperative anxiety and information in perioperative patients

**Figure 2:**
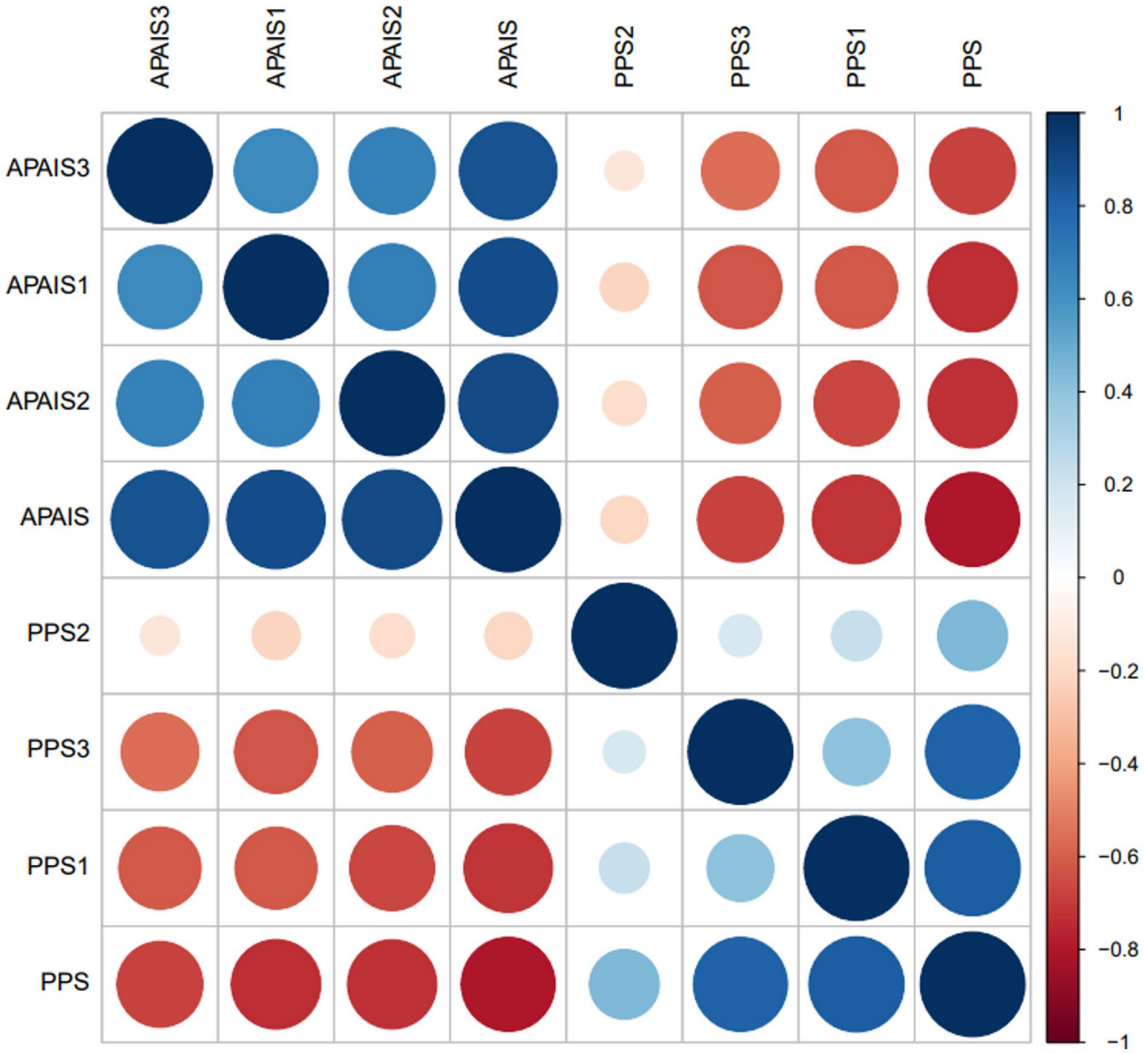
Analysis of the correlation between perioperative patient Amsterdam preoperative anxiety and information scale and privacy satisfaction. PPS, total perioperative patient privacy satisfaction score; PPS 1, preoperative privacy satisfaction score; PPS 2, intraoperative privacy satisfaction score; PPS 3, postoperative privacy satisfaction score; APAIS, total preoperative anxiety and information needs score; APAIS 1, anesthesia-related factors; APAIS 2, surgery-related factors; APAIS 3, information needs factors.

preoperative anxiety and information needs was negatively associated with patient privacy satisfaction (*r* = −0.807, *p* < 0.01) (Figure 2).

### Impact of demographic data on patient privacy perceptions

Defining perioperative patient privacy as the dependent variable, linear regression models all incorporated statistically significant factors and APAIS scores from univariate studies. Table 2 shows the variable assignments.

**Table 2:** Assignment of statistically significant variables and dependent variables in univariate analysis.

Variable	Assignments
Gender	Male =0, Female =1
Age(years)	18–44 = 0, 45–59 = 1, ≥ 60 = 2
Anesthesia modality	General = 0, Local =1, Others =2
Clinic	General surgery =0, Cardiothoracic surgery =1, Orthopedic =2, Urology =3, Others = 4
Number of surgeries(times)	0 = 0, 1 = 1, ≥ 2 = 2
APAIS-C	Original value

Linear regression results showed that gender, age, and APAIS score were influential factors affecting perioperative patient privacy (*p* < 0.05). The details are presented in Table 3.

**Table 3:** Linear regression analysis of perioperative patient privacy.

variable	*β*	SE	*β*^2^	*t*	*p*
Independent variable	73.69	2.54	——	29.06	<0.001
Gender	−2.19	0.74	−0.09	−2.95	0.003
Age(years)	5.13	1.11	0.29	4.63	<0.001
Anesthesia modality	0.35	0.82	0.01	0.43	0.67
Number of surgeries(times)	1.30	0.77	0.05	1.70	0.09
APAIS-C	−1.60	0.06	−0.78	−26.65	<0.001

## Discussion

Currently, the patient privacy scale used in clinical settings was applied to the population of student nurses and nurses (26, 27). It was mainly used to assess the degree of awareness of healthcare professionals regarding the protection of patient privacy. In a survey by Schoopp et al. (28) on the privacy of older adults, it was shown that the level of privacy protection varied significantly across countries, with the UK giving the highest level of privacy protection to patients. A study of patients and healthcare workers in an Indian hospital showed that patients have higher perceptions of privacy than healthcare workers (29). Therefore, medical and nursing staff should pay attention to the degree of perioperative patients’ physiological and psychological privacy protection when conducting medical-related operations (30), and improve the quality and level of medical services.

Some studies have shown that patients in urology and cardiothoracic surgery have a higher need for privacy protection (31, 32), while patients in orthopedics and general surgery have a lower need for privacy protection (33, 34). In this study, the investigators included perioperative patients from different departments to investigate the overall level of satisfaction in the context of the current healthcare environment in China. A multidimensional and holistic study was conducted to understand the level of Chinese patients’ satisfaction with surgical privacy protection in different departments and exposure to different procedures, rather than just investigating perioperative patient satisfaction in specific departments. As the surgical procedure requires full exposure to the surgical field, thus putting the patient’s physical privacy at risk, creating a sense of disrespect, and intensifying the occurrence of doctor-patient conflicts.

In this study, the total perioperative patient score was (53.51 ± 12.54), which is in the low level and similar to the results of previous studies (23). The highest postoperative privacy satisfaction score was recorded. The main reason for this may be related to the fact that patients have received increased attention from the medical staff after surgery and that the medical staff has consciously protected patients’ privacy and reduced the exposure of body parts, so patients’ satisfaction with privacy has increased (35, 36). The study found privacy protection to be a common concern for perioperative patients, suggesting that healthcare professionals should assess, identify and intervene in a timely manner when necessary to improve the quality of the medical visit environment and increase patient well-being during hospitalization.

This study concludes that males, older, and preoperative anxiety and information needs were influential factors affecting patient satisfaction with perioperative privacy. The results of the study showed that gender was an important factor influencing patients’ perioperative privacy satisfaction, which is consistent with the findings of Terán et al. (37). Under the influence of traditional Chinese culture, female patients are more concerned about whether their privacy, such as their bodies and personal information, is exposed during the perioperative period. Therefore, healthcare professionals should take different approaches to provide patients with an integrated and comprehensive sense of inner satisfaction, which will also enable patients to feel more confident in speeding up their disease recovery after surgery.

A strong relationship between age and privacy satisfaction was found among perioperative patients, which is consistent with the results of other studies (38). Patients were of different ages and have different needs for privacy protection. Understanding the perioperative privacy needs of patients of different ages is a prerequisite for continuous improvement of the privacy protection program during surgery so that patients can fully trust the medical and nursing staff and reduce the rate of medical disputes between the two parties. For younger patients, the higher the need for privacy and confidentiality. The medical staff provides as much information as possible about the potential for privacy exposure during the procedure, giving the patient an adequate explanation and thus reducing the occurrence of misunderstandings.

When patients were under general anesthesia during surgery, disadvantages such as unconsciousness and inability to control themselves can put patients in a state of fear and apprehension (39), when they become more sensitive to the degree of privacy exposure and thus privacy satisfaction decreases. It was suggested that medical and nursing staff should pay attention to the protection of privacy for patients under general anesthesia, give patients a full sense of trust, and pay attention to covering with screens when viewing wounds and changing dressings after surgery to provide a comfortable and safe environment for patients and reduce their own psychological burden.

Patients who were undergoing surgery for the first time, are nervous because they are confused about the surgical session (40), thus making the experience less pleasant for the patient. Therefore, medical staff explain the procedure in as much detail as possible to first-time patients, communicate with patients to reduce their own panic, and relax by providing patients with music and other ways to improve privacy satisfaction if necessary.

In this study, preoperative anxiety was at a high level, which is consistent with the findings of Eberhart et al. (41) Patients who are in a state of high mental stress and anxiety in the preoperative period not only affect their anesthesia, but also interfere with the surgical procedure. Effective medical interventions can reduce their anxiety levels (42) and improve the success rate of patients’ surgery. Perioperative patients are more likely to have increased anxiety levels due to their lack of understanding of the surgical process and their worries (43). Therefore, preoperative healthcare professionals should provide effective surgery-related information to alleviate their anxiety levels and increase their knowledge so that they can successfully complete the surgery and have an early recovery.

Patient privacy satisfaction was negatively associated with preoperative anxiety and information needs (*r* = −0.807, *p* < 0.01). The lower the patient’s preoperative anxiety and the lower the information needs, the higher the patient’s privacy satisfaction. This was consistent with the findings of Khorshidi et al. (44). Analyzing the reasons, it is possible that perioperative patients are more concerned about whether their privacy is better protected because of their concerns about the procedure. Preoperative anxiety was a common psychological state for patients, and it had been documented that therapies such as positive stress reduction and relaxation training can alleviate perioperative patient anxiety levels and thus improve privacy satisfaction (45).

## Conclusion

In this study, patient gender, age, and preoperative anxiety and information needs were found to be factors influencing patient privacy satisfaction in the perioperative period, laying the groundwork for subsequent medical staff to provide targeted interventions.

## Limitations

First, the sample in this study was from only one city in northern China, and the different locations may have had some influence on the study results. Secondly, the sample size in this study was limited and could not represent all populations. Therefore, the scope of the study could be expanded in the future to conduct further studies on cultural differences in different countries, different races, different populations, and different disease types.

## Data availability statement

The raw data supporting the conclusions of this article will be made available by the authors, without undue reservation.

## Ethics statement

The studies involving human participants were reviewed and approved by the Ethics Committee of Jinzhou Medical University. The patients/participants provided their written informed consent to participate in this study. Written informed consent was obtained from the individual(s) for the publication of any potentially identifiable images or data included in this article.

## Author contributions

MT: conceptualization and writing–original draft preparation. HL and XW: writing–review and editing. HL: funding acquisitions. All authors have read and agreed to the published version of the manuscript.
